# Protective Effects of a Mixed Medicinal Herb Extract (NUC1) on Collagenase-Induced Osteoarthritis in Rabbits

**DOI:** 10.4014/jmb.2303.03044

**Published:** 2023-07-21

**Authors:** Sung-Gyu Lee, Hyun Kang

**Affiliations:** Department of Medical Laboratory Science, College of Health Science, Dankook University, Cheonan 31116, Republic of Korea

**Keywords:** SW1353, osteoarthritis, matrix metalloproteinase, zymography, collagenase

## Abstract

NUC1 (Nutraceutical compound 1) is an ethanol extract composed of a formulation based on medicinal herbs traditionally used for the treatment of arthritis in Korea and China. This study investigated the therapeutic effects of NUC1 on osteoarthritis (OA). The protective effect of NUC1 on OA was tested in a rabbit model of collagenase-induced arthritis (CIA) for 4 weeks. Results were compared among four groups (*n* = 9 per group): the normal group (untreated), the CIA group (vehicle control), the NUC1 group (CIA rabbits treated with 200 mg/kg NUC1), and the JOINS group (positive control, CIA rabbits treated with 200 mg/kg JOINS tablet). NUC1 significantly inhibited NO production (*p* < 0.05 at 125 μg/ml, *p* < 0.01 at 250 μg/ml, and *p* < 0.001 at 500 μg/ml) and iNOS expression in macrophages, in a concentration-dependent manner. NUC1 also inhibited the release and protein expression of MMP-1, 3, and 13, in TNF-α-induced chondrosarcoma cells in a concentration-dependent manner. In vivo, the MMP-1 and MMP-3 levels in synovial fluids were significantly (*p* < 0.05) lower in NUC1 group (77.50 ± 20.56 and 22.50 ± 7.39 pg/ml, respectively) than in the CIA group (148.33 ± 68.58 and 77.50 ± 20.46 pg/ml, respectively). Also, in histopathological, NUC1 ameliorated articular cartilage damage in OA by increasing the abundance of chondrocytes and proteoglycan in the articular cartilage. Thus, NUC1 showed promise as a potential therapeutic agent, and it can be generalized to a broader study population in different OA animal models.

## Introduction

Osteoarthritis (OA) is the most prevalent type of arthritis, characterized by progressive degradation of articular cartilage in the joints. Connective tissue degeneration is influenced by various factors, including mechanical stress and biochemical changes that disrupt the delicate balance between tissue destruction and regeneration [1]. Articular cartilage acts as a cushion that covers the ends of femurs and tibias to absorb physical shocks to the joint. When exposed to mechanical and chemical stress, articular cartilage wears out, resulting in severe functional deterioration [2]. The pathophysiology of OA primarily involves local inflammation triggered by pro-inflammatory cytokines, such as tumor necrosis factor (TNF)-α and interleukin (IL)-1β, which further induce the upregulation of matrix metalloproteinase (MMP) expression in the articular tissues [3].

Previous studies have indicated that various types of MMPs, such as MMP-1 (collagenase) and MMP-3 (stromelysin), play a significant role in the degradation of cartilage by breaking down extracellular matrix components like collagen and protein polysaccharides. The activities of MMPs are regulated by tissue inhibitors of matrix metalloproteinases (TIMPs), which act as endogenous inhibitors. Both MMPs and TIMPs are known to have crucial involvement in the pathogenesis of OA [4]. In a study conducted by Dean *et al*. [5], cartilage extracts from arthritic samples were analyzed. The findings demonstrated that both MMP and TIMP concentrations were elevated in arthritic samples compared to normal samples, but the increase in MMP concentration was notably higher than the increase in TIMP concentration. These results confirmed that the disproportionate overproduction of MMPs was associated with cartilage destruction.

Currently, OA treatment focuses on the partial relief of symptoms and pain caused by OA-induced inflammation, but it does not specifically treat the disease [6]. Non-steroidal anti-inflammatory drugs (NSAIDs) are an important treatment option for OA symptoms. Although their anti-inflammatory and analgesic effects are effective in relieving OA pain and symptoms, NSAIDs have various side effects, including gastroduodenal ulceration, cardiovascular risks, and prolonged bleeding [7].

NUC1 (Nutraceutical compound 1) is an ethanol extract composed of a formulation based on medicinal herbs traditionally used for the treatment of arthritis in Korea and China [8-11]. This formulation incorporates five exceptional herbs with proven antioxidant and anti-inflammatory properties, as supported by our previous studies [12]. These five herbs are: *Acanthopanax sessiliflorus* (Rupr. & Maxim.) Seem. (*Araliaceae*), *Houttuynia cordata* Thunb. (*Saururaceae*), *Eucommia ulmoides* Oliv. (*Eucommiaceae*), *Kalopanax pictus* (Thunb.) Nakai (*Araliaceae*), and fermented *Achyranthes japonica* (Miq.) Nakai (*Amaranthaceae*). These herbs have been used in oriental medicine for treating arthritis for thousands of years in Korea [13]. In particular, our previous study showed that, among the five herbs, fermented *Achyranthes japonica* (Miq.) Nakai increased the OA treatment effect, because the fermentation technique increased the amount of 20-hydroxyecdysone in the mixture [14].

The current study demonstrated that NUC1 had the following properties: (*i*) it inhibited the production of nitric oxide (NO) in lipopolysaccharide (LPS)-induced macrophages, (*ii*) it lowered the levels of MMPs, such as MMP-1, -3, and -13, in TNF-α induced chondrosarcoma cells, (*iii*) it had protective effects on cartilage destruction of the affected knee joint by influencing MMP levels in an in vivo rabbit model system of collagenase-induced arthritis (CIA). These results suggested that NUC1 showed promise as a potential therapeutic agent that might serve as a chondroprotective drug in OA.

## Materials and Methods

### Preparation of NUC1

All herbs were purchased from a medicinal herb market in Gyeong-dong market, Korea. The medicinal herbs purchased were identified at the Herbal Medicine Resources Research Center, Korea Institute of Oriental Medicine. The herbs had less than 10% moisture content by weight, and they were air-dried. Fermented *A. japonica* (Miq.) Nakai extract was prepared as previously described in detail [14]. We mixed the following herbs together: fermented *A. japonica* (Miq.) Nakai extract (20 g), *A. sessiliflorus* (Rupr. & Maxim.) Seem. (20 g), *H. cordata* Thunb. (20 g), *E. ulmoides* Oliv. (20 g), and *Kalopanax pictus* (Thunb.) Nakai (20 g). The herbal mixture (total 100 g dry weight) was extracted three times with 80% ethanol at 40°C for 3 h in an ultrasonicator. The supernatant was collected by filtration (Whatman No. 2 filter). The extract was concentrated to obtain an ethanol concentrate; then, the concentrated extract was freeze-dried to obtain a powder. Before investigating the effects of NUC1 both in vitro and in vivo, NUC1 was dissolved in phosphate-buffered saline (PBS) for use.

### HPLC Analyses

An optimized high-performance liquid chromatography (HPLC) method was employed to quantify polyphenolic standard compounds simultaneously in NUC1. Caffeic acid, ferulic acid, rutin, quercetin, luteolin, apigenin, and ellagic acid were purchased from Sigma-Aldrich Co. (USA). A standard mixture was prepared at a concentration of 1 mg/ml using a solvent mixture of acetonitrile:methylene chloride (2:3, v/v). The analysis was conducted using an Agilent 1200 series instrument (USA) equipped with a 292 Supelco Discovery C18 column (4.6 mm × 250 mm, 5 μm, MilliporeSigma, USA) as the analytical column. A sample injection volume of 20 μl was used, and the column temperature was maintained at 25°C. Gradient elution was performed using a mobile phase consisting of 3% acetic acid water (A) and acetic acid, acetonitrile, and water (3:25:72, v/v) (B), with a flow rate of 1 ml/min. The gradient elution program was set as follows: 0–5 min, 0% B; 5–40 min, 70% B; 40-45 min, 80% B; 45-55 min, 85%B; 55–57 min, 90% B; 57-75 min, 90% B. The detection wavelength was set at 280 nm.

### Cell Culture and MTT Assays

RAW 264.7 mouse macrophage cells were purchased from the KCLB (Korean Cell Line Bank, Korea). SW 1353 human chondrosarcoma cells were purchased from ATCC (American Type Culture Collection, USA). Cells were grown in DMEM (Dulbecco’s modified Eagle’s medium, Gibco, USA), supplemented with 10% FBS (fetal bovine serum, Gibco) containing penicillin (100 U/ml), and streptomycin (100 μg/ml), in a humidified incubator at 37°C with 5% CO_2_.

We tested the herbal sample for cytotoxic effects on RAW 264.7 and SW 1353 cells with a 3-(4,5-dimethylthiazol-2-yl)-2,5-diphenyl-2H-tetrazolium bromide (MTT) assay [15]. Cells were seeded, respectively, at 1 × 10^5^ and 1 × 10^4^ cells/well in 96-well plates filled with DMEM culture medium. Cells were treated with various concentrations of NUC1 and incubated for the indicated time. Then, a 10 μl of MTT reagent (5 mg/ml) was added to each well, and incubated at 37°C for 2-4 h. The supernatant was then removed, and the formed formazan was dissolved by adding 100 μl of DMSO to all wells. The optical density (O.D.) was measured at 550 nm with a microplate reader (spectra MAX 340 pc, Molecular Devices, USA).

### NO Content Measurement

The seeded RAW 264.7 cells were incubated for 18 h at 37°C cells and treated with different concentrations of NUC1, in the absence (negative control) or presence (positive control) of 100 ng/ml LPS (Sigma). After 24 h of culture, NO concentrations in the cultured medium were determined with the Griess reaction [16]. A 100 μl of SNT (supernatant) from all well was mixed with Griess reagent (100 μl, Sigma) in a separate 96-well plate. After incubating for 10 min at room temperature (RT), the OD was measured at 540 nm with a microplate reader (spectra MAX 340 pc). Nitrite concentrations were determined by comparisons with a standard curve for sodium nitrite (Sigma).

### Casein and Gelatin Zymography

Casein and gelatin zymography were performed according to the procedure described by Gogly *et al*. [17] with minor modifications. SW 1353 cells were seeded in 6-well culture plates at 5 × 10^5^ cells/well, and treated with various concentrations of NUC1 in the presence of 50 ng/ml TNF-α. The culture medium supernatant was electrophoresed through a 10% polyacrylamide gel (contained 0.1% (w/v) casein). The gel was then washed three times with 2.5% Triton X-100. Subsequently, it was incubated at 37°C for 24 to 48 h in a buffer containing 50 mM Tris-HCl (pH 7.5), 10 mM CaCl_2_, and 0.01% NaN_3_. After incubation, it was stained using a 0.2% Coomassie brilliant blue reagent. Proteolysis was detected as a transition from dark blue areas to white areas

### Measurement of MMP-1, MMP-3, and MMP -13 Secretion

SW 1353 cells (1 × 10^4^ cells/well) were seeded into 96-well plates. After 24 h, the medium was removed, and different concentrations of NUC1 were cultured in serum-free DMEM medium along with 50 ng/ml of TNF-α for 24 h. Supernatants were isolated and the levels of MMP-1, MMP-3, and MMP -13 were determined with an ELISA kit (USCN Life Science Inc., UK).

### Western blot analysis

Cell lysis were extracted in RIPA buffer (Thermo Scientific™ RIPA Lysis and Extraction Buffer) containing protein inhibitor (Roche, Germany). Briefly, the cells were incubated for 30 min at 4°C, then centrifuged at 10,000 ×*g* for 15 min; the supernatant was obtained as a cell lysate. Protein concentrations were measured with Bradford dye-binding method. Aliquots of cellular proteins (20 μg/lane) were electrophoresed through a 10%SDS-polyacrylamide gel, then transferred to a 0.45 μM nitrocellulose membrane (Millipore, USA). The membranes were blocked with 5% skim milk for 30 min at RT and then exposed to the primary antibodies at 4°C overnight. The primary antibodies were purchased from Santa Cruz Biotechnology (USA). For secondary antibody incubation, a 1:2,000 dilution of the secondary antibody (Santa Cruz Biotechnology) was used for 2 h at RT.

### Animals and Ethical Statement

New Zealand white male rabbits were housed individually at the animal experimental center at Yeung Nam University College of Medicine (Korea). All experiments involving animals were approved by the Yeung Nam University Institutional Animal Care and Use Committee (YUMC-AEC2010-001, Korea). Animals were housed in quiet environment at 24 ± 1°C temperature and a humidity of 50-60% under a 12 h light/dark cycle the condition of ventilation. They were provided a free access to standard pellet diet and tap water. All the animals were carried out to experiments after one week’s acclimatization. All animals were observed daily by an accredited in laboratory animal scientist, who monitored their health grade and signs of distress. This study was directed by the Guide for the Care and Use of Laboratory Animals (National Institutes of Health Publication No. 85-23).

### The Collagenase-Induced Arthritis Model and NUC1 Treatment

Rabbits weighed 2.5-3.0 kg (*n* = 36, aged 9-10 weeks) at the start of experiments (day 1). For collagenase-induced arthritis (CIA), the rabbits were anesthetized by 0.5 mg/kg tiletamine-zolazepam (Zoletil 50, Virbac Laboratories, France), and the right knee joints were shaved. Next, rabbits received an intra-articular injection of 4 mg/ml type-II collagenase solution (*Clostridium histolyticum*, 425 U/mg, in 0.9% sterile saline) or 250 μl saline (normal control group). The same injection procedure was performed, according to the method described by Kikuchi *et al*. [18].

After the initial collagenase injection (day 1), the rabbits were divided into four groups (*n* = 9 per group): the normal group (untreated), the CIA group (the OA model; considered the vehicle control), the NUC1 group (CIA rabbits treated with 200 mg/kg NUC1), and the JOINS group (positive control, CIA rabbits treated with 200 mg/kg JOINS tablet; SKI306X, SK Chemicals, Korea). JOINS tablet is one of the herbal anti-arthritic drugs. All treatment groups received daily oral administrations of distilled water (normal and CIA groups) or the assigned drug (NUC1 or JOINS groups) for 4 weeks. After 4 weeks, rabbits were euthanized with 0.5 mg/kg tiletamine-zolazepam, and an autopsy was performed.

### Serum Transaminase Levels

Glutamic oxaloacetic transaminase (GOT) and glutamic pyruvic transaminase (GPT) were measured in the sera of CIA rabbits with specific assay kits (USCN Life Science Inc.). GOP and GPT assays were performed at RT, and ODs were measured at 450 nm with a microplate reader (spectra MAX 340 pc).

### MMP-1 and MMP-3 in Synovial Fluid

Synovial fluid was drawn from rabbit knee joints on the day of autopsy to measure MMP-1 and MMP-3 levels. MMP-1 and MMP-3 concentrations were determined with ELISA kits (USCN Life Science Inc., USA) according to the manufacturer's instructions.

### Histopathological Analysis

Rabbits were sacrificed for histological examinations after 4 weeks. The right knee joint was incised, and soft tissues were removed. The lateral and medial aspects of the tibial plateau and femoral condyle were fixed in 10%formalin (pH 7.4) and 10% EDTA for four weeks. The decalcified tissues were placed in 70% ethanol and dehydrated in graded ethanol solutions. Each dehydrated tissues were embedded using paraffin blocks, and 4 μm of serial sections. Specimens were stained with H&E (hematoxylin and eosin) plus toluidine blue. The condition of each articular cartilage sample was scored according to a combination of the OA grade and the OA stage, as described by Pritzker *et al*. [19]. The OA grade was defined as the depth of OA progression into the cartilage.

### Statistical Analysis

The all results are expressed as the mean ± standard deviation (S.D.). Data were analyzed with the analysis of variance (ANOVA). Statistical significance was accepted at *p* < 0.001, compared to the normal group, and at *p* < 0.05, *p* < 0.01, and *p* < 0.001, compared to the negative control group.

## Results

### Phenolic Compound Content in NUC1

**Fig. 1:**
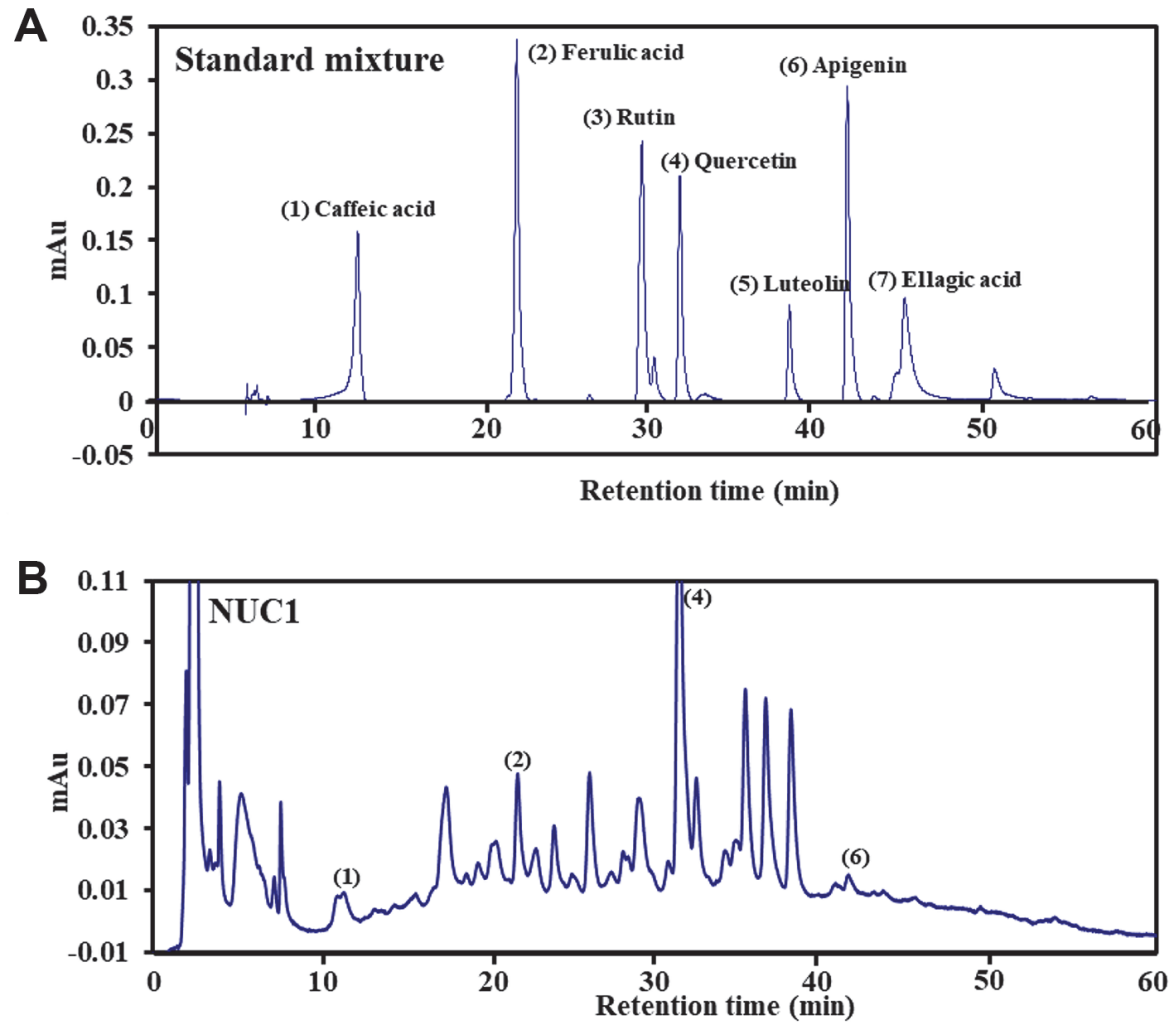
HPLC chromatograms of standard mixture and NUC1. (**A**) The seven standard compounds (1) caffeic acid, (2) ferulic acid, (3) rutin, (4) quercetin, (5) luteolin, (6) apigenin, and (7) ellagic acid were separated by a Discovery C18 analytical column at 25°C at 280 nm. (**B**) HPLC chromatogram of NUC1 (1) caffeic acid, (2) ferulic acid, (4) quercetin, (6) apigenin.

The major phenolic compounds present in NUC1 were identified using HPLC-DAD, as shown in Fig. 1. Analysis of the HPLC chromatogram revealed the presence of four phenolic compounds in NUC1, with the following concentrations: caffeic acid (0.16 mg/g), ferulic acid (0.65 mg/g), quercetin (2.02 mg/g), and apigenin (0.11 mg/g).

### NUC1 Effects on NO Production and iNOS Expression in Macrophages

**Fig. 2:**
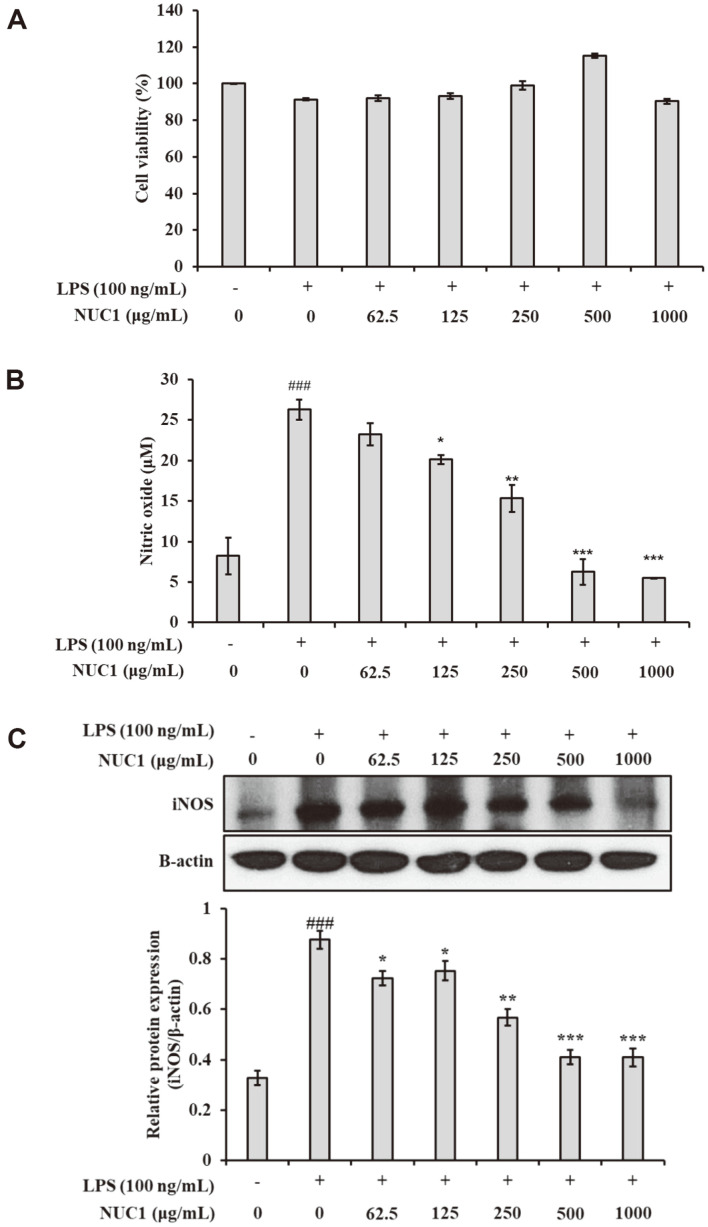
NUC1 effects on LPS-induced NO production and iNOS expression in mouse macrophages. Cells were treated with the indicated concentrations of NUC1 and LPS (100 ng/ml) for 24 h. (**A**) MTT assay results show cell viability. (**B**) Griess assay results indicate the amount of nitrate in supernatants. (**C**) Western blot results show iNOS protein expression; β- actin was used as a loading control. Each value represents the mean ± SD of data obtained from five independent experiments. ^###^*p* < 0.001, compared to the untreated group; *p < 0.05, **p < 0.01, and ***p < 0.001 compared to the group treated with LPS alone.

First, we tested the cytotoxic effects of NUC1 on macrophages with MTT assays. To determine the effective concentration range, we tested NUC1 at concentrations of 62.5, 125, 250, 500, and 1,000 μg/ml respectively. We found that cell viability was over 90% at all concentrations (Fig. 2A). LPS stimulates Toll-like receptors (TLRs) on the surface of macrophages, initiating the activation of the downstream cellular signaling pathway, mitogen-activated protein kinase (MAPK). This activation induces the expression of various pro-inflammatory mediators, including NO, and other inflammatory cytokines. To determine the effects of NUC1 on NO production, we measured the accumulation of nitrite in the culture media with the Griess reagent. We observed the maximum NO production (26.4 μM) after incubating cells with LPS (100 ng/ml) for 24 h. However, NUC1 treatment significantly (*p* < 0.5) inhibited NO production at a concentration of 125 μg/ml (Fig. 2B).

Next, we investigated whether NUC1 suppression of NO production was due to reduced iNOS protein expression. Cells were again stimulated with LPS in the presence or absence of NUC1 at different concentrations for 24 h. The expression of iNOS protein was barely detectable in unstimulated macrophages (Fig. 2C). However, upon LPS treatment, iNOS expression was markedly increased. The addition of NUC1 inhibited iNOS protein expression in LPS-stimulated cells in a concentration-dependent manner.

### NUC1 Effects on MMP-Enzyme Activity in Chondrosarcoma Cells

**Fig. 3:**
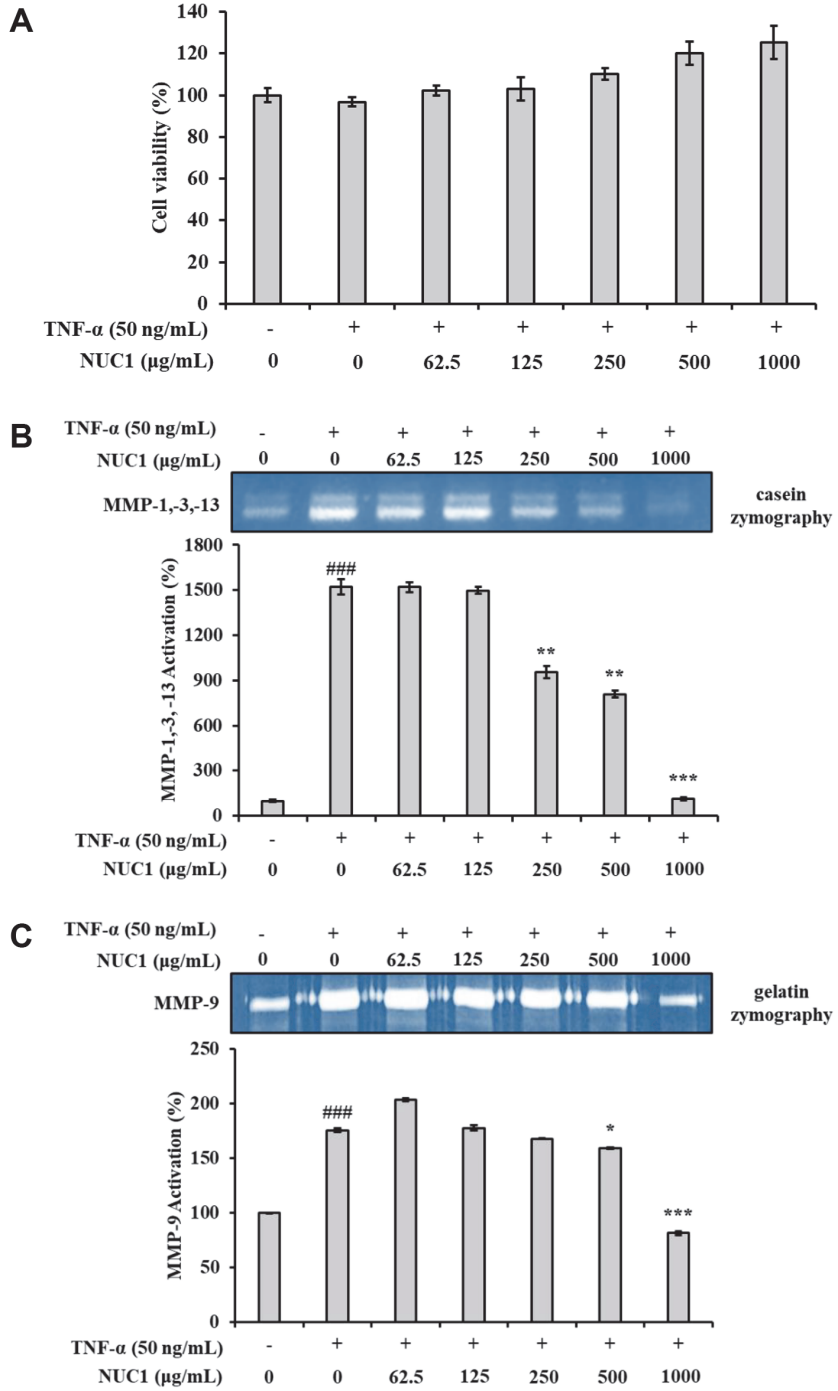
NUC1 Inhibition of TNF-α-induced MMP enzyme activity in human chondrosarcoma cells. Cells were treated with the indicated concentrations of NUC1 and TNF-α (50 ng/ml) for 24 h. (**A**) MTT assay results show cell viability. Each value represents the mean ± SD of data obtained from three independent experiments. (**B**) Casein and (**C**) gelatin zymography results show MMP-1, MMP-3, MMP-9, and MMP-13 activities in culture supernatants. ^###^*p* < 0.001, compared to the untreated group; *p < 0.05, **p < 0.01, and ***p < 0.001 compared to the group treated with TNF-α alone.

The cytotoxicity of NUC1 on chondrosarcoma cells was analyzed with an MTT assay. We found over 90% cell viability in chondrosarcoma cells after 24 h of incubation with NUC1 at concentrations of 62.5, 125, 250, 500, and 1,000 μg/ml (Fig. 3A). Next, we collected cultured media and we evaluated proteolytic MMP activity with casein and gelatin zymography. TNF-α is recognized as a key inflammatory cytokine that functions as a mediator, promoting the excessive production of collagenase by acting on chondrocytes. As shown in Figs. 3B and 3C, MMPs had very weak activity. Treatment with TNF-α (50 ng/ml) increased the level of MMP proteolysis. However, TNF-α-induced MMP activities were inhibited by NUC1 in a concentration-dependent manner.

### NUC1 Effects on MMP-1, MMP-3, and MMP-13 in Chondrosarcoma Cells

**Fig. 4:**
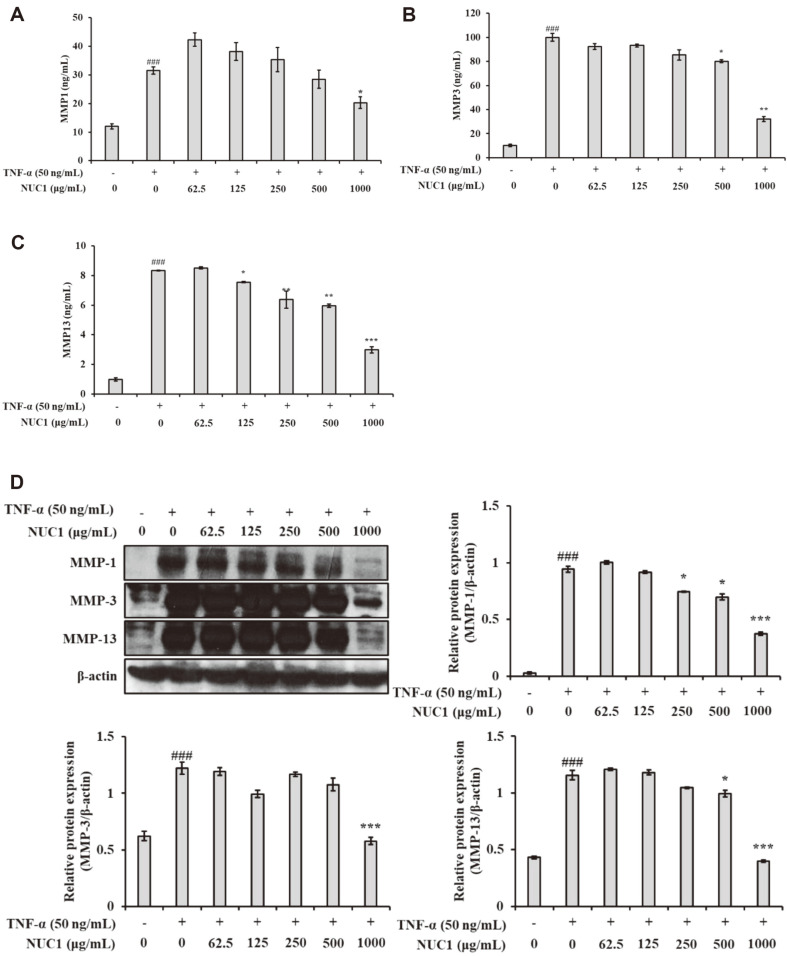
NUC1 effects on TNF-α-induced MMP-1, MMP-3, and MMP-13 release and protein expression in chondrosarcoma cells. Cells were treated with the indicated concentrations of NUC1 and TNF-α (50 ng/ml) for 24 h. ELISA results show the abundance of (**A**) MMP-1, (**B**) MMP-3, and (**C**) MMP-13 in cell supernatants. (**D**) Western blot shows MMP protein expression. β-actin was used as a loading control. Each value represents the mean ± SD of data obtained from three independent experiments. ### *p* < 0.001, compared to the untreated control group; * *p* < 0.05, ** *p* < 0.01, and *** *p* < 0.001, compared to the group treated with TNF-α alone.

Chondrosarcoma cells were treated with different concentrations of NUC1 (0, 62.5, 125, 250, 500, and 1,000 μg/ml) in the presence of TNF-α (50 ng/ml). After 24 h, MMP-1, MMP-3, and MMP-13 release was significantly decreased with NUC1 treatment, compared to TNF-α alone (Figs. 4A-4C). Next, we examined how NUC1 effected the TNF-α-induced expression of specific MMPs. Chondrosarcoma cells were cultured with TNF-α (50 ng/ml) and different concentrations of NUC1. Cell lysates were prepared for western blots to evaluate the specific expression levels of MMP-1 (55 kDa), MMP-3 (57 kDa), and MMP-13 (60 kDa). We found that NUC1 concentrations ranging from 62.5 to 1,000 μg/ml markedly inhibited MMP-1, MMP-3, and MMP-13 protein levels in a dose-dependent manner (Fig. 4D).

### NUC1 Effects on GOT and GPT Activities in a Collagenase-Induced Arthritis Model

**Fig. 5:**
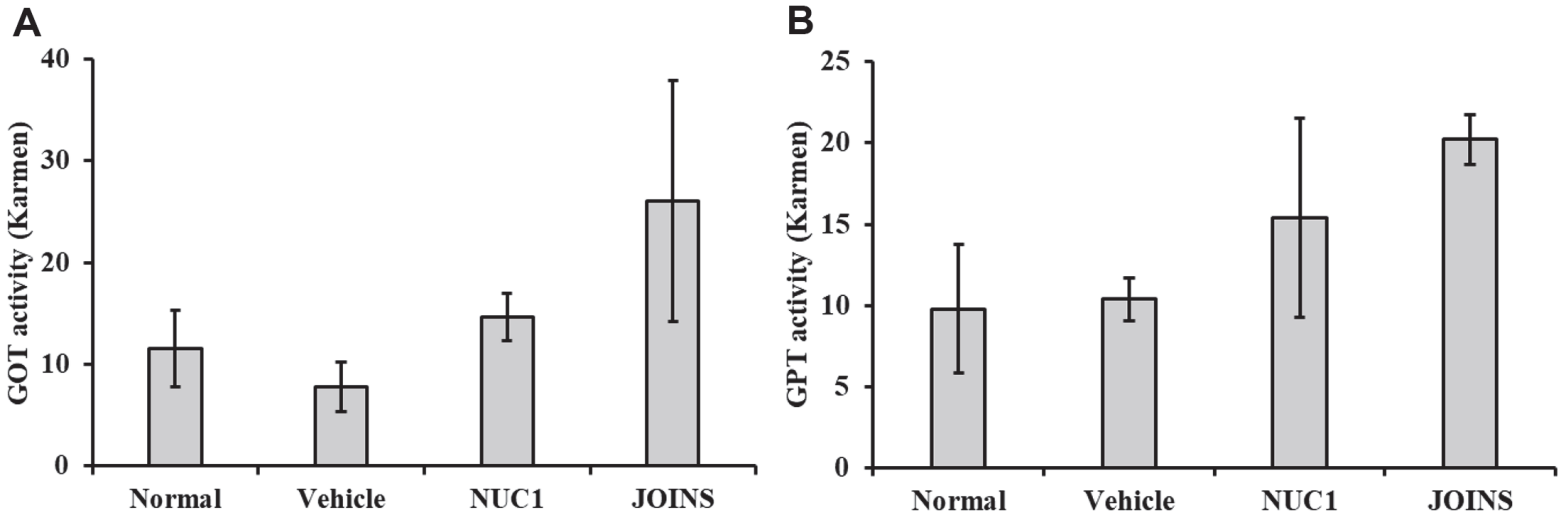
NUC1 effects on serum GOT and GPT levels in CIA rabbit model. ELISA results show the serum levels of (**A**) GOT and (**B**) GPT in four rabbit treatment groups. Each value represents the mean ± SD of data obtained from three independent experiments with 9 rabbits/group. Normal: untreated rabbits; vehicle: CIA rabbits (OA model); NUC1: herbal medicine treatment in CIA rabbits; JOINS: alternative herbal OA treatment in CIA rabbits; CIA: collagenase-induced arthritis; GOT: glutamic oxaloacetic transaminase; GPT: glutamic pyruvic transaminase; OA: osteoarthritis.

We measured the levels of serum markers for liver damage, including GOT and GPT. These enzymes are released into the bloodstream following liver cell damage, with GPT being more specific to liver injury [20]. After measuring the levels of GOT and GPT in each group, we observed a slight increase in the NUC1 and JOINS groups, but there were no significant changes observed among all groups (Fig. 5). The measured values remained within the normal range, indicating no evidence of liver damage caused by the samples.

### NUC1 Effects on MMP-1 and MMP-3 Levels in CIA Rabbits

We examined the effects of NUC1 on the levels of MMP-1 and MMP-3 in the synovial fluid of CIA rabbits. We found that the MMP-1 and MMP-3 levels in synovial fluids were significantly lower in NUC1-treated CIA rabbits than in the CIA alone group (*i.e.*, vehicle control group, Fig. 5). Additionally, the MMP-1 and -3 levels in synovial fluids were significantly lower in the NUC1-treated rabbits than in the JOINS-treated rabbits (*i.e.*, positive control group).

### NUC1 Effects on Histological Features of CIA Rabbits

In the collagenase-induced OA model, the histopathological results of articular cartilage treated with NUC1 are depicted in Fig. 7. In the normal group, the articular cartilage of the knee joint exhibited a smooth surface, and the arrangement of chondrocytes and matrix components was balanced. The knee joints of the normal control group, stained with toluidine blue, displayed rich proteoglycans, appearing as a dense blue color. In contrast, the CIA group showed severe degenerative changes in the articular cartilage, with substantial cartilage loss, pronounced exposure of the calcified zone, and proliferation of surrounding connective tissue. For the NUC1 and JOINS groups, partial exposure of the calcified zone was observed, but the extent of exposure was limited. Additionally, signs of chondrocyte proliferation, indicative of cartilage repair, were observed. Furthermore, compared to the CIA group, a mitigation of proteoglycan loss, which are components of the cartilage matrix, was observed in the NUC1 and JOINS groups. The histopathological observations of the articular cartilage in each experimental group were scored, and the results are showed in Fig. 7B. Compared to the CIA group, both the NUC1 and JOINS groups exhibited significantly (*p < 0.05) reduced degrees of cartilage damage, indicating less severe cartilage destruction. Therefore, NUC1 demonstrated its potential to alleviate cartilage matrix loss and improve OA.

## Discussion

OA is characterized by a progressive loss of articular cartilage, synovitis, inflammation in the synovium, osteophytes, changes to subchondral bone, and growth of new bone and cartilage at the joint edge [3, 21, 22]. In OA, it is known that cartilage destruction is induced by IL-1β and TNF-α proinflammatory cytokines [23]. Recently, it was demonstrated that other mediators, such as prostaglandin E_2_ (PGE_2_) and NO, played important roles in the induction of MMPs in an inflammatory milieu [3]. In addition to the chondrocytes, it was shown that macrophages that infiltrated the OA synovium also contributed to inflammation and matrix degradation in OA tissues [24]. Therefore, inflammatory mediators represent potential targets for OA disease interventions.

NO production is the hallmark of activated macrophages. NO production is mostly catalysed by NOS (nitric oxide synthase), which exists in three different isoforms: endothelial NOS, inducible NOS and neuronal NOS [25]. NO production mediated by iNOS can promote pathological inflammation. Therefore, selective inhibition of iNOS activity has been established as a therapeutic approach for treating inflammation [25]. We found that NUC1 suppressed NO production by inhibiting iNOS protein expression (Fig. 2).

The MMP family of enzymes facilitate cartilage turnover and breakdown. MMP levels are elevated in the joint tissues of patients with OA [26]. Arthritis is considered by the collapse of joint tissues, and it is caused by various factors, including aging, stress, and diabetes [21]. This breakdown can be experimentally mimicked by stimulating chondrocytes with TNF-α, IL-1, or LPS, because these components play prominent roles in the catabolism of articular cartilage [27]. Among the MMPs, MMP-1 and MMP-13 are predominantly important, due to their capability to cleave fibrillar collagen, which is the most plentiful component of the extracellular matrix [28]. Both MMP-1 and -13 have the sole ability to disturb the triple helix structure of collagen [29]. MMP-1 is expressed in fibroblasts, keratinocytes, endothelial cells, monocytes, macrophages, chondrocytes, and osteoblasts [30]. MMP-1 is one of the main enzymes that exactly degrades type II collagen [31]. On the other hand, MMP-13 was shown to be involved in remodeling OA cartilage [32]. In contrast, MMP-3 is a stromelysin formed by stromal cells, with chondrocytes. MMP-3 is up-regulated in the early stage of OA [30]. Both MMP-3 and -13 cleave collagen II and aggrecan. It was shown that human MMP-13 cartilage-specific overexpression of in mice mimicked human OA [33]. Our results showed that NUC1 treatment inhibited the extracellular release of MMPs by blocking TNF-α-induced MMP-1, MMP-3, and MMP-13 expression in chondrosarcoma cells (Fig. 4).

**Fig. 6:**
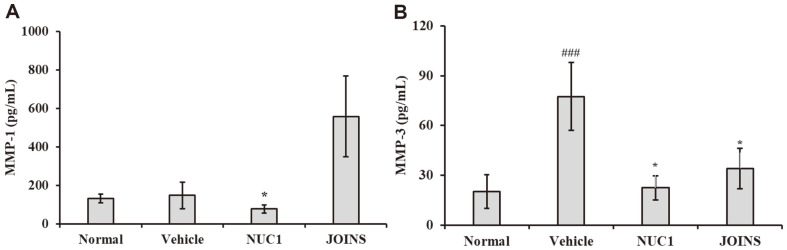
NUC1 effects on MMP-1 and MMP-3 levels in CIA. Synovial fluids were collected from rabbit knee joints and measured with ELISA to determine the activity of (**A**) MMP-1 and (**B**) MMP-3. Each value represents the mean ± SD of data obtained from three independent experiments with 9 rabbits/group. Normal: untreated rabbits; vehicle: CIA rabbits (OA model); NUC1: herbal medicine treatment in CIA rabbits; JOINS: alternative herbal OA treatment in CIA rabbits ^###^*p* < 0.001 compared to normal group; * *p* < 0.05 compared to vehicle group; CIA: collagenase-induced arthritis; OA: osteoarthritis.

**Fig. 7:**
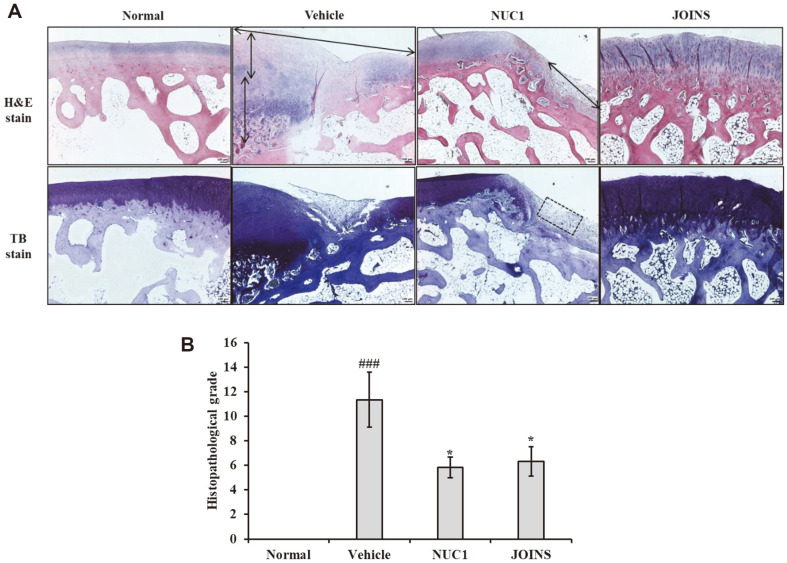
NUC1 protection against cartilage degradation in CIA rabbits. Representative micrographs show articular cartilage samples derived from the right knee joints of rabbits in each treatment group, stained with (**A**) H&E or toluidine blue. (**B**) Histopathological scores based on a combination of the OA grade and the OA stage in the three CIA groups. Each value represents the mean ± SD of data obtained from three independent experiments with 9 rabbits/group. Normal: untreated rabbits; vehicle: CIA rabbits (OA model); NUC1: herbal medicine treatment in CIA rabbits; JOINS: alternative herbal OA treatment in CIA rabbits; ^###^ *p* < 0.001 compared to the normal (untreated) group; * *p* < 0.05 compared to the vehicle (untreated CIA) group; CIA: collagenase-induced arthritis; OA: osteoarthritis.

Articular cartilage is a simple structure composed of cartilage cells surrounded by an extracellular matrix of water, collagen type II, and aggrecan [34]. In OA, cartilage damage is accelerated, due to an imbalance between anabolism and catabolism, which leads to articular cartilage degeneration [35]. Anabolism involves the production of proteoglycans, including aggrecan and collagen type II; catabolism involves the activity of MMPs [30]. Serum and synovial fluid MMP-3 levels were increased in patients with arthritis compared to controls [34]. Another study revealed an association between the synovial fluid MMP-3 concentration at disease onset and the progression of joint destruction [36]. In an in vivo study, NUC1 ameliorated the progress of OA by inhibiting joint destruction. Our findings suggested that this effect could have been due to the regulation of MMP-1 and MMP-3 release by NUC1, which prevented proteoglycan and collagen destruction (Figs. 6 and 7).

Previous studies reported that fermented *A. japonica* (Miq.) Nakai extracts ameliorated OA by inhibiting the production of TNF-α and IL-4 proinflammatory cytokines [37]. In particular, fermentation increased the anti-inflammatory effect by increasing the content of the active component, 20-hydroxyecdysone. The stem bark and root bark of *A. sessiliflorus* (Rupr. & Maxim.) Seem. were reported to have a beneficial effect on OA through its antioxidant and anti-inflammatory properties [8]. Apigenin, the main component of *H. cordata* Thunb., ameliorated damage in a collagen-induced arthritis (CIA) mouse model by repressing synovial hyperplasia, angiogenesis, and osteoclastogenesis [38]. *E. ulmoides* Oliv. is a medicinal herb with a wide variety of active components, including lignan, iridoid, phenol, steroid, flavonoid, and other compounds. Various pharmacological tests have shown that *E. ulmoides* Oliv. had antioxidant, anti-inflammatory, anti-allergic, antibacterial, anticancer, etc. properties, and it has been broadly used alone or in combination with other herbs for considering inflammatory diseases [39]. *K. pictus* (Thunb.) Nakai has significant antinociceptive effects, in addition to its ability to inhibit arthritis. It was suggested that saponins isolated from the stem bark of *K. pictus* (Thunb.) Nakai could inhibit rheumatoid arthritis by inhibiting kinin formation [40]. NUC1 is an ethanol extract from 5 plants, namely fermented *A. japonica* (Miq.) Nakai, *A. sessiliflorus* (Rupr. & Maxim.) Seem., *H. cordata* Thunb., *E. ulmoides* Oliv., and *K. pictus* (Thunb.) Nakai. In this study, HPLC analysis identified four phenolic compounds within NUC1: caffeic acid, ferulic acid, quercetin, and apigenin (Fig. 1). Previous studies have reported the therapeutic and improvement effects of these phenolic compounds on OA [38, 41-43]. Therefore, NUC1, which incorporates these diverse components, is believed to be effective in improving OA.

This study demonstrated that NUC1 inhibited the production of NO in LPS-induced macrophages, and reduced MMP-1, MMP-3, and MMP-13 levels in chondrosarcoma cells treated with TNF-α. Moreover, we showed that NUC1 ameliorated articular cartilage damage in OA by increasing the abundance of chondrocytes and proteoglycan in the articular cartilage. We also found that NUC1 significantly alleviated cartilage degeneration by inactivating MMP-1 and MMP-3. Therefore, NUC1 showed promise as a potential therapeutic agent that might protect against articular cartilage destruction by inhibiting inflammation and MMP activities in OA.
